# A case of secondary syphilis masquerading as cutaneous lymphoma

**DOI:** 10.1016/j.jdcr.2021.05.036

**Published:** 2021-06-09

**Authors:** Kevin Nethers, Rafael E. Mojica, Etan Marks, Robin Burger, Sadia Saeed, William Steffes

**Affiliations:** a Kansas City University-Graduate Medical Education Consortium/Advanced Dermatology and Cosmetic Surgery Orlando Dermatology Residency Program, Oviedo, Florida; b Edward Via College of Osteopathic Medicine, Department of Graduate Medical Education, Spartanburg, South Carolina; c Advanced Dermatology and Cosmetic Surgery, Department of Graduate Medical Education, Maitland, Florida

**Keywords:** cutaneous T-cell lymphoma, lymphoma mimicker, secondary syphilis, T-cell gene rearrangement positivity

## Introduction

Classically, secondary syphilis presents as a generalized nonpruritic papulosquamous eruption, which may include the palms and soles, typically occuring 3 to 10 weeks after the initial spirochete inoculation.^1^^,^^2^ Numerous unusual presentations have been reported, including but not limited to, annular or figurative plaques, “moth-eaten” alopecia, polymorphic papules, granulomatous nodules, crusted nodules, and nail changes.^3^ Typical histologic findings include a superficial and deep perivascular lymphocytic infiltrate with a variable admixture of plasma cells. Histological findings of syphilis, however, can also vary widely and may mimic other disease states.^4^

## Case report

**Fig 1:**
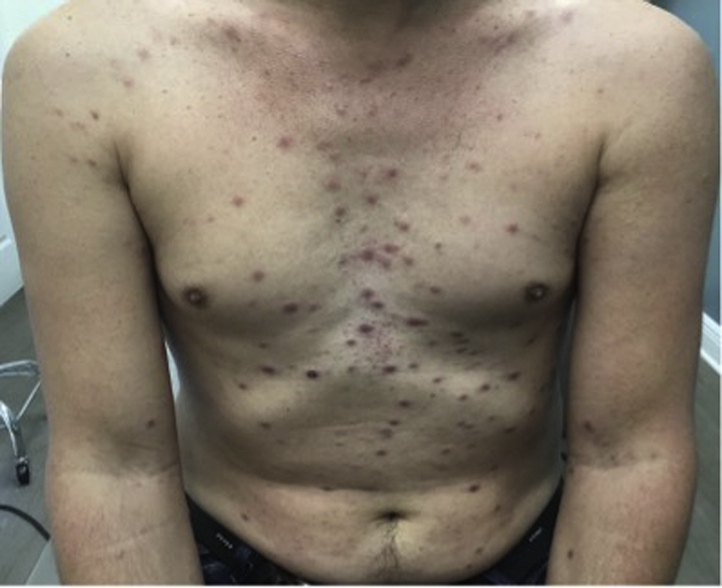
Widespread red-brown papulovesicles on the trunk and upper extremities at initial presentation.

**Fig 2:**
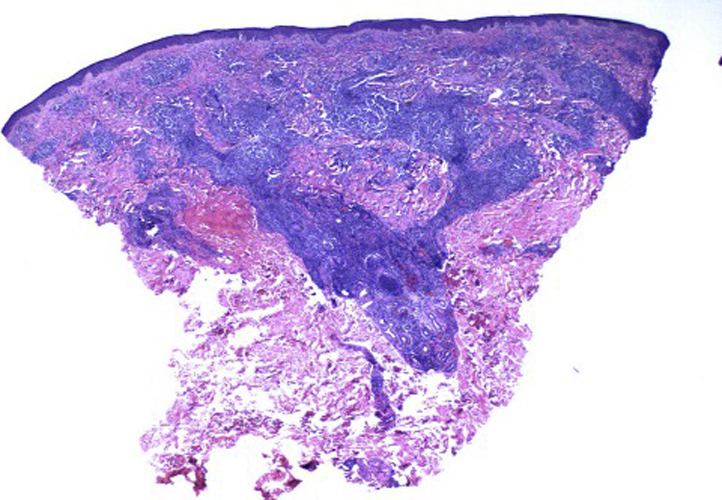
A hematoxylin-eosin–stained punch biopsy collected at initial presentation. Dense superficial and deep perivascular and periadnexal dermal infiltrates comprising mostly small lymphocytes, some medium-sized lymphocytes, and a few plasma cells and histiocytes.

We present a 44-year-old Caucasian man with crops of red-brown papulovesicles on his trunk and upper extremities, which had been present for 2 weeks (Fig 1). Palms and soles were not involved. Lymphadenopathy was not present. He denied pruritus, fevers, chills, purulent drainage, or any other current symptoms. He denied a history of chancre or new sexual partners. His past medical history included hypertension and hyperlipidemia. He was currently taking amlodipine, gemfibrozil, hydrochlorothiazide, and simvastatin. Although the differential was broad, the clinical presentation was concerning for lymphomatoid papulosis. Punch biopsies of the epigastric skin and left ventral forearm lesions were performed. Both specimens demonstrated a dense superficial and deep perivascular and periadnexal dermal infiltrate comprised mostly of small lymphocytes, some medium-sized lymphocytes, and a few plasma cells and histiocytes (Fig 2). Immunoperoxidase analysis of the infiltrates showed predominantly CD20/CD79a^+^ B-cells, associated with a background of CD3^+^ T cells with a normal CD4:8 ratio. There was variable bcl-2 reactivity, while bcl-6 and cyclin D1 were negative. CD30 immunoperoxidase analysis was also negative. Both biopsies showed polyclonal results via kappa and lambda in situ hybridization studies.

Given the dense lymphocytic infiltrate on microscopy and the clinical concern for cutaneous lymphoma, both specimens were submitted for T- and B-cell gene rearrangement studies. The epigastric specimen exhibited positive T-cell receptor beta and gamma gene rearrangements. The forearm specimen exhibited negative T-cell receptor beta gene rearrangement; however, the biopsy was positive for T-cell receptor gamma gene rearrangement (demonstrating the same clonal pattern as that observed in the epigastric skin biopsy). B-cell clonality studies were negative for both specimens. These molecular findings raised concern for a T-cell lymphoproliferative disorder, although a reactive clonal expansion could not be completely ruled out. Further workup included an oncology consultation and subsequent dermatology follow up. Medical therapy was not initiated at this time, as the patient was asymptomatic, and as the diagnosis was still in question. Early blood work revealed an unremarkable complete blood count, comprehensive metabolic panel, and protein electrophoresis.

**Fig 3:**
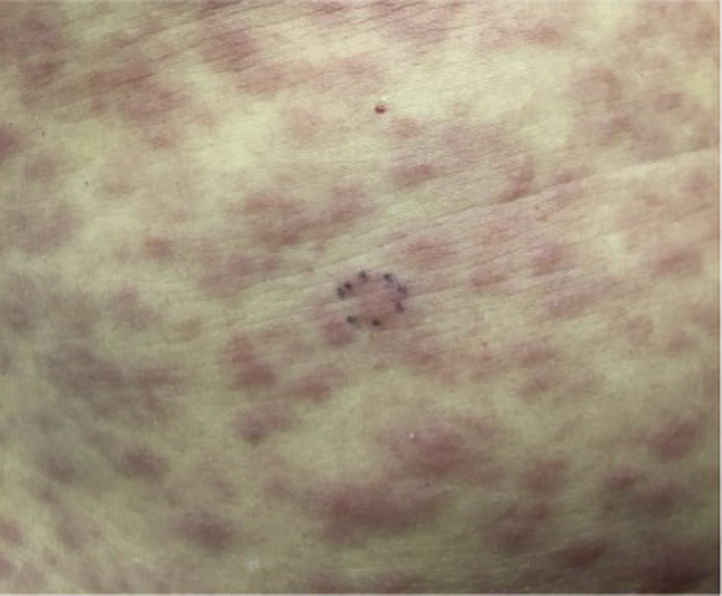
A close-up look of the generalized papulosquamous rash 7 weeks after initial presentation.

**Fig 4:**
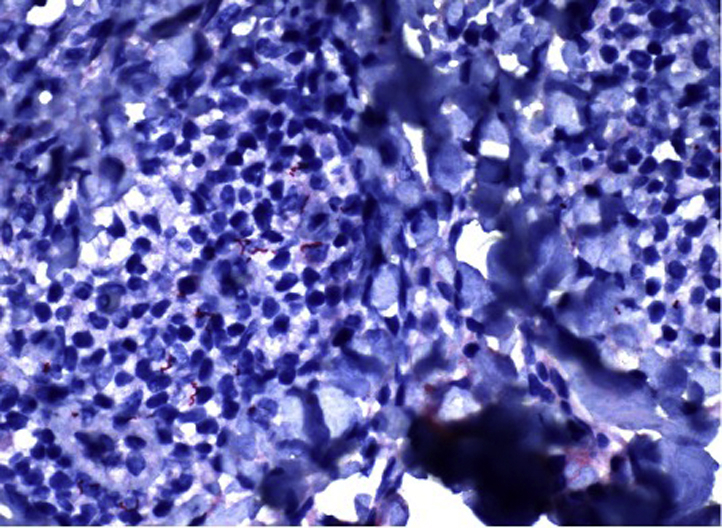
Spirochetes present in the dermal layer of biopsy specimen submitted for immunohistochemical analysis.

The oncology team in conjunction with the dermatology team requested additional biopsies prior to initiating any therapy. Upon the follow-up visit with dermatology, 7 weeks from the initial visit, the patient showed a more generalized papulosquamous rash (Fig 3) on the trunk and extremities (but not palms and soles). A re-biopsy was performed, which showed mild epidermal hyperplasia, with a less dense lymphoplasmacytic perivascular and periadnexal dermal infiltrate. *Treponema pallidum* immunoperoxidase analysis revealed spirochetes in the dermis, supporting a diagnosis of secondary syphilis (Fig 4). In addition, *T. pallidum* immunoperoxidase analysis was performed in retrospect, in the previous 2 biopsies, and both exhibited spirochetes. HIV and hepatitis panels were negative. A rapid plasma reagent test was positive at a titer of 1:128, and a fluorescent treponemal antibody absorption test was also positive.

The patient was sent to the health care department for further therapy. After antibiotic therapy with 28 days of oral doxycycline, the eruption resolved. Lymphoproliferative disorders were ruled out given the prompt resolution of the eruption, clarity on secondary biopsies, and lack of recurrence at the 6-month follow-up. The identical T-cell clonality in the 2 initial biopsies was attributed to a reactive process.

## Discussion

Syphilis has been widely documented as mimicking other diseases clinically as well as histologically; however, syphilis mimicking cutaneous lymphoma/lymphoreticular disease has seldomly been reported.^1^, ^2^, ^3^^,^^5^^,^^6^,^7^ The majority of these cases are HIV positive patients (Table I).^1^^,^^5^, ^6^, ^7^ Braue et al^7^ reported a unique case of rupioid syphilis in an HIV patient, which initially mimicked cutaneous lymphoma. Similar to our case, early biopsies in that case revealed atypical lymphohistiocytic infiltrate, and the T-cell receptor gene was clonally rearranged. Lymph-node and bone-marrow biopsies failed to show evidence of lymphoma. Upon follow up, subsequent skin biopsies showed an atypical lymphohistiocytic infiltrate, which appeared to be reactive without clonal etiology. Flow cytometry of the skin specimen showed predominantly small mature lymphocytes and histiocytes with no clonal findings and no evidence of T-cell lymphoma. Fluorescent treponemal antibody absorption and rapid plasma reagent were positive. The diagnosis of rupioid syphilis was made. Notably, his eruption worsened greatly during initiation of anti-retrovial therapy but subsequently resolved with penicillin.

**Table I:** Summary of previous reports of syphilis mimicking cutaneous lymphoma

Age/sex	38 years/man	37 years/man	40s/man	36 years/man	38 years/man
Reference	Laungani et al^1^	McComb et al^2^	Yamashita et al^5^	Braue et al^7^	Glover et al^6^
Initial presentation	Fevers, chills, and disseminated nonpuritic erythematous papules, which began in the abdominal area without involvement of the palm and soles.	No systemic symptoms. Non-pruritic erythematous macules, thin plaques and papules in the upper trunk, cheeks, and upper eyelids.	Fever, headaches, arthralgia, weight loss, and pruritic skin macules over the trunk and extremities.	Fevers, night sweats, and yellowish-brown, necrotic crusted malodorous verrucous plaques in the scalp, nose, legs, and arms shortly after starting anti-retroviral therapy.	Malaise and chills. Nontender, nonpruritic macules on the anterior aspect of the thighs, trunk, and extremities, sparing the palms and soles. Shiny indurated plaques in the scalp, eyebrows, and eyelashes.
HIV status	Positive	Negative	Positive	Positive	Positive
Cutaneous Histology	Atypical lymphohistiocytic infiltrate at the dermo-epidermal junction, exocytosis of atypical lymphocytes, and minimal spongiosis.	Dense lymphohistiocytic infiltrate in the mid and reticular dermis; perivascular infiltrates of plasma cells, neutrophils, and scattered monocytes; inflammatory infiltrates extending to the subcutaneous fat.	Epidermotropism of atypical lymphoid cells with venulitis and extravasation of blood. Neutrophils and histiocytes were abundant; limited number of plasma cells and eosinophils.	Atypical lymphohistiocytic infiltrate consisting of CD3^+^, CD7^+^, and CD8^+^ T cells with clonal T-cell receptor rearrangement. Reactive atypical lymphohistiocytic infiltrate on subsequent skin biopsy.	Thinning of the epidermis with focal areas of erosion and crust. Inflammatory infiltrate was also found containing, lymphocytes, histiocytes and plasma cells.
Work up	Patient misdiagnosed as having cutaneous T-cell lymphoma based on initial skin biopsy findings and false-negative RPR result. Progression of papules to involve the palms and soles, and a repeat positive RPR test result followed by a positive FTA-ABS test result confirmed the correct diagnosis of secondary syphilis.	A diagnosis of cutaneous T-cell lymphoma was suspected based on cell marker studies and histologic findings but could not be established due to the absence of the expected monotypic light chain expression. It was the presence of plasma cells in the infiltrate that led to the workup and diagnosis of secondary syphilis.	Based on skin biopsy findings and a proliferation of CD8^+^ CD30- T cells, the authors quoted the WHO classification for tumors to provide support their diagnosis of cutaneous peripheral T-cell lymphoma. An analysis of the bone marrow for tumor staging purposes revealed that up to 10% of all nuclear cells were atypical lymphocytes, an abnormal finding that the authors interpreted as a reactive change due to secondary syphilis. This new diagnosis was supported by immunohistochemical staining for *Treponema* on the dermis layer.	Based on initial skin biopsy findings and a normal lymphoid population identified by flow cytometry, the initial suspected diagnosis was cutaneous peripheral T-cell lymphoma not otherwise specified. Subsequent skin biopsy led to the workup of secondary syphilis with both FTA-ABS and RPR tests being positive. This presentation was attributed to rupioid syphilis in the setting of immune reconstitution syndrome secondary to antiretroviral therapy.	Initial skin histopathology and clinical presentation led to the differential diagnoses of mycoses fungoides, cutaneous T-cell lymphoma, and leukemia cutis. Further workup including a positive VDRL test led to the diagnosis and treatment of secondary syphilis.

*FTA-ABS*, Fluorescent treponemal antibody absorption; *RPR*, rapid plasma reagent; *VDRL*, venereal disease research laboratory; *WHO*, World Health Organization.

As of 2003, only 8 cases of secondary syphilis had been reported to histologically mimic a malignant neoplasm.^2^ In review of these cases provided by McComb et al,^2^ a variety of findings were present including a dense lymphohistiocytic infiltrate in the mid and reticular dermis, perivascular infiltrates composed of histiocytes, plasma cells, neutrophils and scattered monocytes with hyperchromatic nuclei and prominent nucleoli, mitotic figures, epidermal hyperkeratosis, mild acanthosis, focal microabscesses, and inflammatory infiltrates extending to the subcutaneous fat. T-cell receptor gene rearrangement studies were negative when tested.

Herein, we report a case of secondary syphilis with matching monoclonal T-cell receptor gene rearrangements from multiple biopsy specimens in an HIV negative immunocompetent patient. Typically, an infectious process, such as syphilis, would cause a reactive type of polyclonality, but there are cases of transient reactive T-cell monoclonality in patients with infections. This has been reported with Epstein-Barr virus,^8^ cytomegalovirus infections and the above mentioned rupioid syphilis case.^7^^,^^9^ In all these cases, T-cell lymphoma was the leading diagnosis until seroconversion and symptomology changes occurred.

In our case, concomitant disease states or syphilis led initiation of cutaneous lymphoproliferative disorder were considered. There are links between certain infectious/inflammatory processes and lymphoproliferative diseases. This is evident in disease states such as chronic atopic dermatitis proceeding cutaneous T-cell lymphoma and Merkel cell polyoma virus proceeding Merkel cell carcinoma.^10^ Syphilis, however, has not typically been reported to precede malignant processes and we do not foresee this happening in our patient as the symptoms resolved with antibiotic therapy.

## Conflicts of interest

None declared.
